# Altered Raphe-Centered Monoamine Transporter Covariance in Early Parkinson’s Disease with Depressive Symptoms

**DOI:** 10.3390/diagnostics16152318

**Published:** 2026-07-24

**Authors:** Yeongjoo Lee, Seunggyun Ha, Sang-Won Yoo, Joo Hyun O, Ie Ryung Yoo, Joong-Seok Kim

**Affiliations:** 1 Division of Nuclear Medicine, Department of Radiology, Seoul St. Mary’s Hospital, College of Medicine, The Catholic University of Korea, Seoul 06591, Republic of Korea; soap2222@cmcnu.or.kr (Y.L.); ojoohyun@songeui.ac.kr (J.H.O.); iryoo@catholic.ac.kr (I.R.Y.); 2 Department of Neurology, Seoul St. Mary’s Hospital, College of Medicine, The Catholic University of Korea, Seoul 06591, Republic of Korea; hiswy83@gmail.com (S.-W.Y.); neuronet@catholic.ac.kr (J.-S.K.)

**Keywords:** Parkinson disease, depression, monoamine transporter, covariance analysis, positron emission tomography

## Abstract

**Background/Objectives**: Depression is a common non-motor manifestation of early Parkinson’s disease (PD), but its underlying neurobiological mechanisms remain incompletely understood. Although alterations in serotonergic pathways have been implicated, conventional molecular imaging studies have primarily focused on regional tracer uptake and have yielded inconsistent findings. We investigated whether raphe-centered monoamine transporter covariance and metabolic covariance are altered in early PD with depressive symptoms. **Methods**: Patients with early PD underwent bimodal PET imaging using extrastriatal [^18^F]FP-CIT PET as a putative marker of raphe-centered monoamine transporter–related organization and early-phase [^18^F]florbetaben PET as a surrogate of regional cerebral perfusion. Group-level covariance analyses were performed using the dorsal and median raphe nuclei as seed regions, and covariance patterns were compared between depressive and non-depressive PD groups. Regional tracer uptake was also evaluated. **Results**: Compared with the non-depressive group, patients with depressive PD demonstrated significantly greater dorsal raphe-associated monoamine transporter covariance involving the left precuneus, left inferior parietal lobule, and right cuneus after false discovery rate correction. In contrast, no significant between-group differences were identified for median raphe-associated monoamine transporter covariance or raphe-associated metabolic covariance after multiple-comparison correction. Regional extrastriatal [^18^F]FP-CIT uptake and early-phase [^18^F]florbetaben perfusion were also comparable between groups. **Conclusions**: Depressive symptoms in early PD were associated with altered dorsal raphe-centered monoamine transporter covariance in the absence of detectable regional imaging abnormalities. Group-level covariance analysis may complement conventional regional PET analyses for investigating the network-level neurobiology of depression in PD.

## 1. Introduction

Parkinson’s disease (PD) is a common neurodegenerative disorder characterized not only by motor symptoms but also by a wide range of non-motor manifestations. Among these, depression is one of the most prevalent and clinically impactful complications, significantly impairing the quality of life of patients with PD [1,2,3]. Previous studies have reported that depression affects up to 50% of patients with PD, encompassing both major and minor depressive episodes [4,5]. Despite its high prevalence and clinical impact, the neural mechanisms underlying depression in PD remain incompletely understood, particularly at the level of distributed brain networks.

Alterations in the serotonergic system have long been implicated in the pathophysiology of depression. The dorsal raphe (DR) and median raphe (MR) nuclei are the principal sources of ascending serotonergic projections. While the DR provides the major serotonergic innervation to widespread cortical and subcortical regions, including the amygdala involved in mood regulation, the MR preferentially projects to limbic and hippocampal structures and also contributes to emotional and cognitive processing [6,7]. In PD, neuropathological and molecular imaging studies have demonstrated degeneration of serotonergic neurons within the raphe nuclei and abnormalities of serotonin transporter availability, suggesting that disruption of serotonergic pathways may contribute to depressive symptoms [8,9]. However, in vivo imaging studies examining regional serotonin transporter availability have yielded inconsistent results, and the relationship between regional serotonergic alterations and depression in PD remains incompletely understood [10,11]. Notably, a recent PET study using the highly selective SERT tracers [^11^C]DASB and [^11^C]MADAM demonstrated preserved regional SERT availability in patients with early nondepressed PD [12]. Together, these findings underscore the complexity of serotonergic alterations in PD.

Although N-3-[^18^F]fluoropropyl-2β-carbomethoxy-3β-4-iodophenyl nortropane ([^18^F]FP-CIT) was originally developed as a dopamine transporter tracer, its extrastriatal uptake has been used as a surrogate marker of serotonin transporter–related signal because dopamine transporter expression is relatively sparse outside the striatum [13]. Nevertheless, extrastriatal [^18^F]FP-CIT does not selectively quantify serotonergic terminals and likely reflects mixed monoamine transporter binding. Considering that depression in PD is widely recognized as a multifaceted condition involving alterations across various monoaminergic systems rather than isolated serotonergic deficits [14], extrastriatal [^18^F]FP-CIT uptake may nevertheless provide information relevant to broader monoamine transporter-related alterations. Accordingly, throughout the present study, extrastriatal [^18^F]FP-CIT is interpreted as a putative marker of raphe-centered monoamine transporter–related organization rather than a selective measure of serotonergic integrity. This interpretation is consistent with previous molecular imaging studies investigating non-motor manifestations of PD using extrastriatal FP-CIT imaging [15]. However, conventional molecular imaging studies have focused on regional tracer uptake, which may overlook coordinated alterations across distributed brain networks. Group-level covariance analysis provides a complementary framework for characterizing coordinated interregional variations in tracer uptake across individuals, thereby enabling the investigation of network-level organization beyond conventional regional analyses.

Based on this rationale, we investigated whether depressive symptoms in early PD are associated with altered raphe-centered monoamine transporter covariance using extrastriatal [^18^F]FP-CIT PET. We also evaluated raphe-centered metabolic covariance using early-phase [^18^F]florbetaben ([^18^F]FBB) PET to characterize metabolic network alterations associated with depressive symptoms.

## 2. Materials and Methods

### 2.1. Subjects

We retrospectively reviewed the medical records of patients with early Parkinson’s disease (PD) who underwent both brain [^18^F]FP-CIT PET/CT (FutureChem Co., Ltd., Seoul, Republic of Korea) and [^18^F]FBB PET/CT (Life Molecular Imaging GmbH, Berlin, Germany; distributed by DuChemBio Co., Ltd., Seoul, Republic of Korea) at Seoul St. Mary’s Hospital between December 2018 and September 2020. Among 129 patients diagnosed with idiopathic PD, 22 were excluded because early-phase [^18^F]FBB (eFBB) PET/CT was unavailable, leaving 107 patients with paired [^18^F]FP-CIT PET/CT and eFBB PET/CT datasets.

Clinical variables, including age, sex, years of education, disease duration, modified Hoehn and Yahr (mH&Y) stage, Korean Mini-Mental State Examination (K-MMSE), Clinical Dementia Rating (CDR), Global Deterioration Scale (GDS), Unified Parkinson’s Disease Rating Scale (UPDRS), Geriatric Depression Scale (SGDS), and current antiparkinsonian medications, were collected from the electronic medical records.

**Figure 1:**
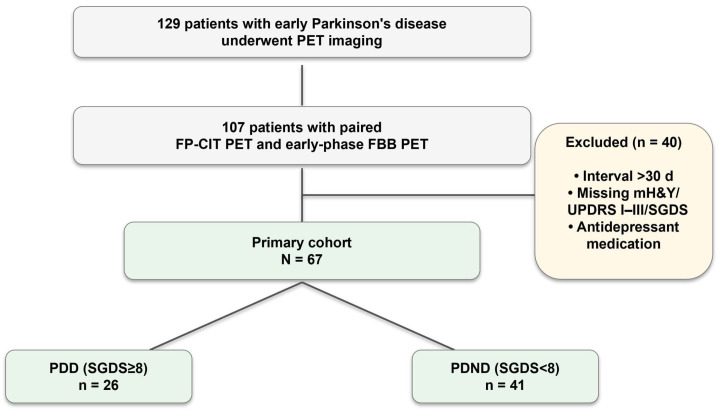
Study flow diagram. Flowchart illustrating patient selection and application of the inclusion and exclusion criteria.

To minimize the potential influence of temporal changes between imaging modalities, the primary analysis was restricted to patients whose interval between the two PET examinations was ≤30 days. Patients with missing clinical variables required for group classification or covariate adjustment, including the short form of the SGDS, mH&Y stage, or UPDRS Parts I–III, were also excluded. To minimize pharmacologic effects on monoaminergic systems, patients with a history of antidepressant use were excluded. Patients receiving antiparkinsonian medications were eligible for inclusion, provided that no clinically significant changes in medication had occurred before PET imaging. Because some patients met more than one exclusion criterion, the final study cohort comprised 67 patients. The study flow diagram illustrating patient enrollment, application of the eligibility criteria, and the final study cohort is shown in Figure 1.

Patients were classified into the Parkinson’s disease with depressive symptoms (PDD) and Parkinson’s disease without depressive symptoms (PDND) groups using an SGDS cutoff of ≥8, based on prior validation studies [16,17,18]. This study was approved by the Institutional Review Board of Seoul St. Mary’s Hospital (IRB No.: KC22RASI0192; approval date: 31 March 2022), and the requirement for informed consent was waived due to the retrospective nature of the study.

### 2.2. Image Acquisition and Reconstruction

Static [^18^F]FP-CIT PET/CT scans were acquired using two PET/CT systems (Biograph 40 TruePoint, Siemens Healthineers, Knoxville, TN, USA; Discovery PET/CT 710, GE Healthcare, Waukesha, WI, USA). Following intravenous administration of [^18^F]FP-CIT (189–218 MBq), a static brain PET acquisition was performed for 10 min at approximately 3 h post-injection [19]. A low-dose CT scan was acquired for attenuation correction prior to PET acquisition. PET images were reconstructed using a 3D ordered-subsets expectation maximization (OSEM) algorithm with a 256 × 256 matrix and a 3 mm Gaussian post-reconstruction filter. Reconstruction parameters were as follows: 6 iterations and 16 subsets for the Biograph system, and 4 iterations and 16 subsets (Vue Point HD) for the Discovery system. Despite differences in reconstruction parameters, standardized preprocessing and intensity normalization were applied in subsequent analyses to mitigate inter-scanner variability.

All [^18^F]FBB PET/CT scans were acquired using the Discovery PET/CT 710 system. For early-phase imaging, a 10 min static PET acquisition was performed immediately after tracer injection (296–326 MBq), corresponding to the perfusion phase. A low-dose CT scan for attenuation correction was acquired prior to PET acquisition. Delayed-phase [^18^F]FBB PET imaging was performed 90 min post-injection, with a 20 min static acquisition. Both early- and delayed-phase images were reconstructed using a 3D OSEM algorithm (4 iterations, 16 subsets; Vue Point HD), with a 256 × 256 matrix and a 3 mm Gaussian filter, consistent with the [^18^F]FP-CIT reconstruction framework. Delayed-phase [^18^F]FBB PET was visually interpreted by experienced readers (Y.L. and S.H.) to determine amyloid positivity by consensus, which was included as a covariate.

### 2.3. Brain PET Preprocessing and Quantification

PET image preprocessing was performed using Statistical Parametric Mapping 8 software (SPM8; Wellcome Trust Centre for Neuroimaging, University College London, London, UK). For spatial normalization to Montreal Neurological Institute (MNI) space, each PET image was co-registered to the corresponding T1-weighted magnetic resonance imaging (MRI) using a rigid transformation. Spatially normalized PET images were smoothed with an isotropic Gaussian kernel of 8 mm full width at half maximum to improve signal-to-noise ratio and accommodate inter-individual anatomical variability.

For quantitative analysis, intensity normalization was performed using standardized uptake value ratios (SUVRs). For [^18^F]FP-CIT PET, SUVR was calculated using a composite occipital reference region derived from the Automated Anatomical Labeling atlas version 3 (AAL3) [20]. Intensity normalization of early phase [^18^F]FBB (e[^18^F]FBB) PET scan was carried out by calculating SUVR_eFBB_, with the reference region being the whole cerebellum template as defined by the Centiloid project [21]. The normalized PET images were parcellated by the extrastriatal regions of interest (ROIs) using the AAL3 atlas, which includes the dorsal and median raphe (MR) [22]. Detailed ROI information used in the analysis is presented in Supplementary Table S1.

### 2.4. Seed-Based Raphe-Associated Covariance Analysis

To characterize raphe-associated covariance, the dorsal raphe (DR) and median raphe (MR) nuclei were defined as seed ROIs. Raphe-associated covariance was operationally defined as the group-level inter-subject covariance between the raphe nuclei and extrastriatal cortical regions, rather than subject-specific functional or metabolic connectivity. Accordingly, the resulting covariance measures characterize coordinated inter-individual variation in tracer uptake at the population level and are not intended to represent individual connectivity measures or to be correlated directly with clinical variables. Because this covariance-based approach relies on inter-subject variability, it does not imply directional or causal interactions between brain regions.

For the PDD and PDND groups, raphe-associated covariance was quantified by calculating Spearman’s rank partial correlation coefficients between the mean standardized uptake value ratio (SUVR) of each extrastriatal ROI and that of the corresponding seed ROI across subjects. Partial correlation was used to estimate group-level covariance while accounting for potential confounding effects of demographic and disease-related variables.

DR- and MR-associated metabolic covariance (MC) was derived from early-phase [^18^F]FBB PET, which reflects regional perfusion as a surrogate marker of cerebral metabolic activity. DR- and MR-associated monoamine transporter covariance (MTC) was assessed using extrastriatal [^18^F]FP-CIT PET uptake as an index of monoamine transporter-related signal. In the primary analyses, partial correlations were adjusted for age, sex, modified Hoehn and Yahr (mH&Y) stage, and binary amyloid deposition status.

To evaluate the robustness of the findings, two sensitivity analyses were additionally performed by further adjusting the partial correlation models for (1) UPDRS Part III alone and (2) UPDRS Parts II and III, thereby accounting for potential confounding effects of motor impairment and motor-related disability.

This covariance-based approach yields a measure of population-level covariance between the raphe nuclei and extrastriatal cortical regions, reflecting the extent to which inter-individual variability in seed uptake is associated with variability in target-region uptake across subjects.

### 2.5. Statistics

All statistical analyses were performed using SPSS version 24.0 (IBM Corp., Armonk, NY, USA) and MATLAB R2021b (MathWorks, Natick, MA, USA).

Clinical and demographic variables were compared between the PDD and PDND groups. Continuous variables were compared using the Mann–Whitney U test, and categorical variables were compared using Fisher’s exact test.

Group differences in regional and voxel-wise SUVR values derived from [^18^F]FP-CIT PET and early-phase [^18^F]FBB PET were assessed using ROI-based analysis and voxel-wise analysis in SPM8. For these analyses, multiple comparisons were controlled using the false discovery rate (FDR) procedure with a significance threshold of q < 0.05.

Group differences in raphe-associated MC and MTC were evaluated using permutation-based comparisons of Spearman’s rank partial correlation coefficients with 20,000 randomizations. Partial correlations were adjusted for age, sex, mH&Y stage, and binary amyloid deposition status. Statistical significance was determined empirically by comparing the observed between-group difference in partial correlation coefficients with the null distribution generated from the permutations.

Because covariance analyses were performed separately for the DR and MR seed regions, multiple comparisons across target ROIs were controlled using the Benjamini–Hochberg FDR procedure within each seed, with statistical significance defined as q < 0.05.

Sensitivity analyses were additionally performed by further adjusting the partial correlation models for (1) UPDRS Part III alone and (2) UPDRS Parts II and III to evaluate the robustness of the observed covariance patterns.

Unless otherwise specified, all statistical tests were two-sided, and a *p*-value < 0.05 was considered statistically significant.

## 3. Results

### 3.1. Demographics

Of the 67 patients included in the primary analysis, 26 were classified as PDD and 41 as PDND according to the predefined SGDS cutoff. The median interval between the two PET examinations was 2 days (range, 1–30 days).

The clinical characteristics of the two groups are summarized in Table 1. The PDD and PDND groups did not differ significantly in age, sex, disease duration, years of education, mH&Y stage, cognitive measures (K-MMSE, GDS, and CDR), or amyloid positivity. Compared with the PDND group, the PDD group also had significantly higher UPDRS Part I and Part II scores, whereas total UPDRS score and UPDRS Part III score were not significantly different.

At the time of clinical assessment, 51 of the 67 patients (76.1%) were naïve to dopaminergic medications (levodopa and dopamine agonists). No significant between-group differences were observed in the use of levodopa, dopamine agonists, monoamine oxidase-B inhibitors, cholinesterase inhibitors, or anxiolytics (Supplementary Table S2).

### 3.2. Voxel-Wise and Regional SUVR Comparison

Voxel-wise analysis using SPM revealed no significant differences in extrastriatal SUVR between the PDD and PDND groups on either [^18^F]FP-CIT or early-phase [^18^F]FBB PET after FDR correction (all q > 0.05). Consistent with the voxel-wise findings, ROI-based analyses also showed no significant group differences in extrastriatal SUVR values. Similarly, no significant between-group differences were observed in the SUVR of DR or MR on either [^18^F]FP-CIT PET or early-phase [^18^F]FBB PET (Table 2).

### 3.3. Raphe-Associated MTC

Group differences in raphe-associated MTC were evaluated using permutation-based comparisons of Spearman’s rank partial correlation coefficients derived from extrastriatal [^18^F]FP-CIT uptake.

For the DR seed, the PDD group demonstrated significantly stronger MTC than the PDND group in the left precuneus (Δρ = 0.895, q = 0.038), left inferior parietal lobule (Δρ = 0.868, q = 0.038), and right cuneus (Δρ = 0.856, q = 0.038) after Benjamini–Hochberg false discovery rate correction (Table 3). Several additional cortical regions showed nominal between-group differences (uncorrected *p* < 0.05) but did not survive correction for multiple comparisons.

For the MR seed, no significant between-group differences in MTC were identified before or after FDR correction.

Overall, altered raphe-associated MTC in PDD was characterized by increased DR-associated covariance involving posterior parietal and occipital cortical regions, whereas MR-associated covariance remained comparable between the two groups.

### 3.4. Raphe-Associated MC

Group differences in raphe-associated metabolic covariance (MC) were evaluated using permutation-based comparisons of Spearman’s rank partial correlation coefficients derived from early-phase [^18^F]florbetaben PET uptake.

For the dorsal raphe (DR) seed, several regions demonstrated nominal between-group differences in MC (uncorrected *p* < 0.05), including the right superior anterior cingulate cortex, and right middle cingulate cortex. However, none of these differences remained significant after Benjamini–Hochberg false discovery rate (FDR) correction (all q > 0.05).

Similarly, for the median raphe (MR) seed, several regions exhibited nominal between-group differences, including the right inferior orbital frontal gyrus, right middle cingulate cortex, and right anterior cingulate cortex, but none survived FDR correction (all q > 0.05).

Overall, no significant between-group differences in raphe-associated MC were identified after correction for multiple comparisons.

### 3.5. Sensitivity Analysis

To evaluate the robustness of the primary findings, the covariance analyses were repeated after additionally adjusting for motor symptom severity. Two sensitivity models were examined by including either UPDRS Part III or UPDRS Parts II and III as additional covariates.

For raphe-associated MTC, the overall spatial pattern remained largely unchanged in both sensitivity analyses (Table S3). The significant DR-associated covariance differences identified in the primary analysis were preserved after adjustment for UPDRS Part III, with four regions remaining significant after FDR correction: the left precuneus, right precuneus, left inferior parietal lobule, and right cuneus. After additional adjustment for UPDRS Parts II and III, significant group differences persisted in the left precuneus, right precuneus, and right cuneus, whereas the association in the left inferior parietal lobule no longer survived FDR correction. No significant MR-associated covariance differences were observed in either sensitivity analysis.

In contrast, raphe-associated MC remained non-significant after additional adjustment for motor symptom severity. No DR- or MR-associated metabolic covariance differences survived FDR correction in either sensitivity model.

These findings indicate that the observed alterations in raphe-associated MTC were largely independent of motor symptom severity, whereas no robust group differences were identified for raphe-associated MC.

## 4. Discussion

In this study, we investigated group differences in raphe-associated MTC and MC in patients with early PD with and without depressive symptoms using multimodal PET imaging. The principal findings were as follows. First, despite the absence of significant group differences in regional extrastriatal [^18^F]FP-CIT uptake or early-phase [^18^F]florbetaben perfusion, patients with depressive symptoms exhibited significantly stronger dorsal raphe-associated MTC involving the left precuneus, left inferior parietal lobule, and right cuneus. Second, these MTC alterations remained largely unchanged after additional adjustment for motor symptom severity, supporting the robustness of the observed covariance patterns. Third, in contrast to MTC, no significant group differences were identified in raphe-associated MC after correction for multiple comparisons. Together, these findings suggest that depression in early PD is associated with alterations in monoamine transporter-related network organization rather than regional tracer uptake or metabolic covariance.

A notable finding of the present study is that altered raphe-associated MTC was detected despite the absence of significant regional differences in extrastriatal [^18^F]FP-CIT uptake or raphe SUVR. This suggests that depression-related alterations in early PD may be reflected more prominently in the organization of monoamine transporter-related covariance than in regional tracer uptake itself. Unlike regional SUVR, which quantifies tracer accumulation within individual brain regions, covariance analysis captures coordinated inter-individual variation between anatomically distinct regions and may therefore be more sensitive to subtle alterations in large-scale brain organization. Importantly, these covariance measures should not be interpreted as direct evidence of anatomical or functional connectivity, but rather as indices of population-level network organization derived from inter-subject covariance. Furthermore, because extrastriatal [^18^F]FP-CIT uptake reflects mixed monoaminergic transporter binding rather than selective serotonin transporter availability, the observed covariance patterns should be interpreted as alterations in monoamine transporter-related network organization rather than selective serotonergic dysfunction [15]. These findings indicate that network-level covariance analysis may reveal disease-related alterations that are not detectable using conventional regional uptake analyses alone.

The spatial distribution of altered MTC may provide further insights into the neural substrates of depressive symptoms in early PD. The significant covariance differences were predominantly localized to posterior association cortices, including the precuneus, inferior parietal lobule, and cuneus. These posterior association cortices serve as major hubs within large-scale brain networks supporting self-referential processing, attentional control, and other higher-order cognitive functions [23,24,25]. The precuneus serves as a central hub of the default mode network and plays a key role in self-referential processing, whereas the inferior parietal lobule is involved in attentional reorienting and higher-order cognitive integration. Although the functional significance of the observed increases in MTC remains uncertain, they may reflect network-level reorganization of monoamine transporter-related brain networks rather than localized alterations in monoamine transporter availability. Because these covariance differences were identified in the absence of regional uptake abnormalities, they are more likely to represent alterations in coordinated network organization than localized changes in tracer uptake.

Our findings are also consistent with accumulating neuroimaging evidence suggesting that depression in PD is associated with disruption of distributed brain networks rather than isolated regional abnormalities. Resting-state functional MRI studies have consistently demonstrated altered functional connectivity within and between the default mode, frontoparietal control, salience, and limbic networks in patients with PD and depressive symptoms [26,27,28]. Notably, several of these studies implicated the precuneus and inferior parietal lobule, key nodes of the default mode and frontoparietal networks, in the pathophysiology of PD-related depression. While these previous investigations characterized network dysfunction using functional MRI, our findings extend this concept by demonstrating altered monoamine transporter-related covariance measured with PET. Although covariance analysis does not directly measure anatomical or functional connectivity, the convergence of findings across imaging modalities supports the notion that altered network organization is a fundamental feature of depressive symptoms in early PD.

The use of the raphe nuclei as seed regions provides important biological context for the present findings. The dorsal raphe nucleus is the principal source of ascending serotonergic projections and serves as a key hub of raphe-centered monoaminergic systems that modulate widespread cortical and subcortical networks involved in mood regulation, cognitive processing, and large-scale brain network organization. Dysfunction of raphe-centered circuits has long been implicated in the pathophysiology of depression, including depression associated with PD. In this context, the altered covariance patterns originating from the dorsal raphe may reflect disturbances in the organization of raphe-centered monoaminergic systems. However, because extrastriatal [^18^F]FP-CIT exhibits mixed affinity for monoamine transporters and does not selectively quantify serotonergic terminals, the present findings do not permit a specific interpretation in terms of isolated serotonergic dysfunction. Rather, the observed covariance patterns may reflect alterations in raphe-centered monoaminergic systems, of which the ascending serotonergic system likely represents a major, but not exclusive, component.

Several limitations should be acknowledged. First, this was a cross-sectional study, precluding conclusions regarding the temporal evolution of monoamine transporter covariance or its causal relationship with depressive symptoms. Longitudinal studies are needed to determine whether alterations in raphe-centered monoaminergic systems precede the development of depression or evolve in parallel with disease progression.

Second, covariance analysis was performed at the group level and reflects inter-subject covariance rather than anatomical or functional connectivity at the individual level. Consequently, the observed covariance patterns should be interpreted as markers of coordinated network organization rather than direct measures of neural connectivity.

Third, depressive symptoms were assessed using the SGDS, a validated screening instrument rather than a structured psychiatric diagnostic interview. Therefore, misclassification of depression status cannot be completely excluded. Nevertheless, the SGDS has demonstrated good agreement with established depression scales, including the Montgomery–Åsberg Depression Rating Scale (MADRS; *r* = 0.780) and the 9-item Patient Health Questionnaire (PHQ-9; *r* = 0.766) [29]. Furthermore, an SGDS cutoff score of 8 has been reported to provide good sensitivity (0.8548) and specificity (0.6957) for major depression in older adults [16].

Fourth, although the dorsal raphe was selected as the seed region based on its established role as the major source of ascending serotonergic projections, extrastriatal [18F]FP-CIT exhibits mixed affinity for monoamine transporters. Therefore, the biological interpretation of the observed covariance patterns should be considered within the broader context of raphe-centered monoaminergic systems rather than selective serotonergic dysfunction.

Fifth, although we adjusted for major clinical covariates, including age, sex, modified Hoehn and Yahr stage, and UPDRS scores, residual confounding by other non-motor symptoms, such as anxiety, apathy, sleep disturbance, autonomic dysfunction, or cognitive impairment, cannot be completely excluded.

Finally, our cohort consisted of patients with early PD, and the findings may not be directly generalizable to more advanced disease stages or other parkinsonian disorders.

Despite these limitations, the present study demonstrates that covariance analysis can detect network-level abnormalities that are not apparent on conventional regional uptake analysis, providing complementary information on raphe-centered monoaminergic organization in early PD.

## 5. Conclusions

In conclusion, depressive symptoms in early Parkinson’s disease were associated with altered monoamine transporter covariance originating from the dorsal raphe, despite the absence of significant regional differences in extrastriatal [^18^F]FP-CIT uptake. These alterations were predominantly observed in posterior association cortices and may reflect disrupted organization of raphe-centered monoaminergic systems. Collectively, these findings demonstrate that group-level covariance analysis provides a complementary framework for investigating monoamine transporter-related network organization in early Parkinson’s disease beyond conventional regional uptake analysis.

## Figures and Tables

**Table 1:** Clinical characteristics of patients.

Variables	PDD (SGDS ≥ 8)	PDND (SGDS < 8)	*p*-Value
Number	26	41	-
Sex (Male:Female)	15:11	28:13	0.606
Age, years	69 [54–86]	71 [50–86]	0.443
Disease duration (years)	1.3 ± 1.3	1.4 ± 1.2	0.896
Education years	10.3 ± 4.3	11.0 ± 4.7	0.590
SGDS	11 [8~15]	4 [0~7]	<0.001 *
UPDRS total	39.0 ± 26.8	26.7 ± 12.9	0.146
Part 1	5.3 ± 6.0	2.4 ± 2.8	0.012 *
Part 2	9.8 ± 7.7	5.8 ± 3.7	0.028 *
Part 3	24.2 ± 15.2	19.1 ± 9.9	0.246
mH&Y stage	1.8 ± 0.8	1.7 ± 0.7	0.652
MMSE	26.0 ± 4.7	26.8 ± 2.8	0.795
Global Deterioration Scale	2.8 ± 0.8	2.7 ± 0.5	0.799
CDR	0.42 ± 0.27	0.38 ± 0.21	0.532
Amyloid (positive:negative)	3:23	7:34	0.729

Note: Data are presented as mean ± standard deviation unless otherwise indicated. Age and SGDS are presented as median (range). *, statistically significant. Abbreviations: CDR, Clinical Dementia Rating; mH&Y, modified Hoehn & Yahr; MMSE, Mini-Mental State Examination; SGDS, Short form of Geriatric Depression Scale; UPDRS, Unified Parkinson’s Disease Rating Scale.

**Table 2:** Comparison of raphe SUVR between the depressive and non-depressive PD groups.

PET/CT Scan	ROI Name	Depressive	Non-Depressive	*p*-Value
[^18^F]FP-CIT	DR	1.65 ± 0.118	1.63 ± 0.14	0.576
	MR	1.56 ± 0.151	1.57 ± 0.19	0.933
e[^18^F]FBB	DR	1.06 ± 0.053	1.05 ± 0.06	0.183
	MR	1.06 ± 0.058	1.06 ± 0.07	0.593

**Table 3:** Regions showing significant group differences in raphe-associated MTC.

Seed	Brain Region	Δρ (PDD − PDND)	Permutation *p*	BH-FDR q	Direction
DR	Left inferior parietal lobule	0.868	0.0007	0.0384	Stronger in PDD
DR	Left precuneus	0.895	0.0009	0.0384	Stronger in PDD
DR	Right cuneus	0.856	0.0012	0.0384	Stronger in PDD

Note: Only regions surviving seed-wise Benjamini–Hochberg false discovery rate correction (q < 0.05) are presented. Observed differences represent covariance in the PDD group minus that in the PDND group.

## Data Availability

The data that support the findings of this study are available from the corresponding author upon reasonable request. The data are not publicly available due to privacy and ethical restrictions involving patient information.

## Supplementary Materials

The following supporting information can be downloaded at https://www.mdpi.com/article/10.3390/diagnostics16152318/s1, Table S1: Extrastriatal ROIs used in the research from AAL3 atlas; Table S2: Medication status; Table S3: Sensitivity analysis.

## Abbreviations

The following abbreviations are used in this manuscript:

PD	Parkinson’s disease
PDD	Parkinson’s disease with depressive symptoms
PDND	Parkinson’s disease without depressive symptoms
DR	Dorsal raphe
MR	Median raphe
[^18^F]FBB	[^18^F]florbetaben
[^18^F]FP-CIT	N-3-[^18^F]fluoropropyl-2β-carbomethoxy-3β-4-iodophenyl nortropane
MC	Metabolic covariance
MTC	Monoamine transporter covariance
CDR	Clinical Dementia Rating
mH&Y	Modified Hoehn & Yahr
MMSE	Mini-Mental State Examination
SGDS	Short form of Geriatric Depression Scale
UPDRS	Unified Parkinson’s Disease Rating Scale
ROI	Region of interest
