# Supplementary material for: Altered Raphe-Centered Monoamine Transporter Covariance in Early Parkinson’s Disease with Depressive Symptoms
Source: Diagnostics (Basel). 2026 Jul 24;16(15):2318. doi: 10.3390/diagnostics16152318 (PMC13465064; doi:10.3390/diagnostics16152318)
Supplement: Supplementary file 1 [file diagnostics-16-02318-s001.zip › diagnostics-4352139-supplementary.pdf]

[Supplementary Materials]

Altered Raphe-Centered Monoamine Transporter Covariance in Early Parkinson's Disease with Depressive Symptoms

Yeongjoo Lee[1], Seunggyun Ha[1,*], Sang-Won Yoo[2], Joo Hyun O[1], Ie Ryung Yoo[1], Joong-Seok Kim[2]

[1] Division of Nuclear Medicine, Department of Radiology, Seoul St. Mary's Hospital, College of Medicine, The Catholic University of Korea; e-mail@e-mail.com
[2] Department of Neurology, Seoul St. Mary's Hospital, College of Medicine, The Catholic University of Korea; e-mail@e-mail.com
***** Correspondence: Seunggyun.ha@catholic.ac.kr; Tel.: +82-2-2258-1547

Supplementary Table S1. Extrastriatal ROIs used in the research from AAL3 atlas

No.	ROI numbering in AAL3 atlas	Hemisphere	Lobes or region	ROI Name
1	1	L	Frontal lobe	Precentral gyrus
2	2	R	Frontal lobe	Precentral gyrus
3	3	L	Frontal lobe	Superior frontal gyrus, dorsolateral
4	4	R	Frontal lobe	Superior frontal gyrus, dorsolateral
5	5	L	Frontal lobe	Middle frontal gyrus
6	6	R	Frontal lobe	Middle frontal gyrus
7	7	L	Frontal lobe	Inferior frontal gyrus, opercular part
8	8	R	Frontal lobe	Inferior frontal gyrus, opercular part
9	9	L	Frontal lobe	Inferior frontal gyrus, triangular part
10	10	R	Frontal lobe	Inferior frontal gyrus, triangular part
11	11	L	Frontal lobe	IFG pars orbitalis,
12	12	R	Frontal lobe	IFG pars orbitalis,
13	13	L	Frontal lobe	Rolandic operculum
14	14	R	Frontal lobe	Rolandic operculum
15	15	L	Frontal lobe	Supplementary motor area
16	16	R	Frontal lobe	Supplementary motor area
17	17	L	Frontal lobe	Olfactory cortex
18	18	R	Frontal lobe	Olfactory cortex
19	19	L	Frontal lobe	Superior frontal gyrus, medial
20	20	R	Frontal lobe	Superior frontal gyrus, medial
21	21	L	Frontal lobe	Superior frontal gyrus, medial orbital
22	22	R	Frontal lobe	Superior frontal gyrus, medial orbital
23	23	L	Frontal lobe	Gyrus rectus
24	24	R	Frontal lobe	Gyrus rectus
25	25	L	Frontal lobe	Medial orbital gyrus
26	26	R	Frontal lobe	Medial orbital gyrus
27	27	L	Frontal lobe	Anterior orbital gyrus
28	28	R	Frontal lobe	Anterior orbital gyrus
29	29	L	Frontal lobe	Posterior orbital gyrus
30	30	R	Frontal lobe	Posterior orbital gyrus
31	31	L	Frontal lobe	Lateral orbital gyrus
32	32	R	Frontal lobe	Lateral orbital gyrus
33	33	L	Insula	Insula

34	34	R	Insula	Insula
35	151	L	Cingulate	Anterior cingulate cortex, subgenual
36	152	R	Cingulate	Anterior cingulate cortex, subgenual
37	153	L	Cingulate	Anterior cingulate cortex, pregenual
38	154	R	Cingulate	Anterior cingulate cortex, pregenual
39	155	L	Cingulate	Anterior cingulate cortex, supracallosal
40	156	R	Cingulate	Anterior cingulate cortex, supracallosal
41	37	L	Cingulate	Middle cingulate & paracingulate gyri
42	38	R	Cingulate	Middle cingulate & paracingulate gyri
43	39	L	Cingulate	Posterior cingulate gyrus
44	40	R	Cingulate	Posterior cingulate gyrus
45	41	L	Limbic system	Hippocampus
46	42	R	Limbic system	Hippocampus
47	43	L	Limbic system	Parahippocampal gyrus
48	44	R	Limbic system	Parahippocampal gyrus
49	45	L	Limbic system	Amygdala
50	46	R	Limbic system	Amygdala
51	47	L	Occipital lobe	Calcarine fissure and surrounding cortex
52	48	R	Occipital lobe	Calcarine fissure and surrounding cortex
53	49	L	Occipital lobe	Cuneus
54	50	R	Occipital lobe	Cuneus
55	51	L	Occipitotemporal area	Lingual gyrus
56	52	R	Occipitotemporal area	Lingual gyrus
57	53	L	Occipital lobe	Superior occipital gyrus
58	54	R	Occipital lobe	Superior occipital gyrus
59	55	L	Occipital lobe	Middle occipital gyrus
60	56	R	Occipital lobe	Middle occipital gyrus
61	57	L	Occipital lobe	Inferior occipital gyrus
62	58	R	Occipital lobe	Inferior occipital gyrus
63	59	L	Occipitotemporal area	Fusiform gyrus
64	60	R	Occipitotemporal area	Fusiform gyrus
65	61	L	Parietal lobe	Postcentral gyrus
66	62	R	Parietal lobe	Postcentral gyrus
67	63	L	Parietal lobe	Superior parietal gyrus
68	64	R	Parietal lobe	Superior parietal gyrus
69	65	L	Parietal lobe	Inferior parietal gyrus, excluding supramarginal and angular gyri

70	66	R	Parietal lobe	Inferior parietal gyrus, excluding supramarginal and angular gyri
71	67	L	Parietal lobe	SupraMarginal gyrus
72	68	R	Parietal lobe	SupraMarginal gyrus
73	69	L	Parietal lobe	Angular gyrus
74	70	R	Parietal lobe	Angular gyrus
75	71	L	Parietal lobe	Precuneus
76	72	R	Parietal lobe	Precuneus
77	73	L	Parietal lobe	Paracentral lobule
78	74	R	Parietal lobe	Paracentral lobule
79	83	L	Temporal lobe	Heschl's gyrus
80	84	R	Temporal lobe	Heschl's gyrus
81	85	L	Temporal lobe	Superior temporal gyrus
82	86	R	Temporal lobe	Superior temporal gyrus
83	87	L	Temporal lobe	Temporal pole: superior temporal gyrus
84	88	R	Temporal lobe	Temporal pole: superior temporal gyrus
85	89	L	Temporal lobe	Middle temporal gyrus
86	90	R	Temporal lobe	Middle temporal gyrus
87	91	L	Temporal lobe	Temporal pole: middle temporal gyrus
88	92	R	Temporal lobe	Temporal pole: middle temporal gyrus
89	93	L	Temporal lobe	Inferior temporal gyrus
90	94	R	Temporal lobe	Inferior temporal gyrus
91	169	-	Midbrain	Raphe nucleus, dorsal
92	170	-	Midbrain	Raphe nucleus, median

Supplementary Table S2. Medication status

Medication	PDD n (%)	PDND n (%)	p value
Levodopa	4 (15.4)	12 (29.3)	0.247
Dopamine agonist	4 (15.4)	8 (19.5)	0.761
MAO-B inhibitor	1 (3.8)	1 (2.4)	1.000
Cholinesterase inhibitor	0 (0.0)	1 (2.4)	1.000
Anxiolytic/Sedative	1 (3.8)	3 (7.3)	1.000
Antipsychotic	0 (0.0)	1 (2.4)	1.000

Supplementary Table S3. Sensitivity analysis

	Primary model Covariates: age, sex, mH&Y, amyloid			Sensitivity model 1 Covariates: age, sex, UPDRS part 3, amyloid			Sensitivity model 2 Covariates: age, sex, UPDRS part 2, UPDRS part 3, amyloid		
ROI Name	Δρ (PDD-PDND)	Raw p	FDR q	Δρ (PDD-PDND)	Raw p	FDR q	Δρ (PDD-PDND)	Raw p	FDR q
Parietal_Inf_L	0.868	0.0007	0.0384	0.738	0.0018	0.0456	0.738	0.0028	0.0672
Precuneus_L	0.895	0.0009	0.0384	0.897	0.0003	0.0240	0.965	0.0002	0.0192
Cuneus_R	0.856	0.0012	0.0384	0.788	0.0019	0.0456	0.798	0.0009	0.0288
Precuneus_R	0.740	0.0072	0.1728	0.858	0.0005	0.0240	0.891	0.0005	0.0240

Only regions surviving seed-wise Benjamini–Hochberg false discovery rate correction ($q < 0.05$) are presented. Observed differences represent covariance in the PDD group minus that in the PDND group. No median raphe-associated monoamine transporter covariance findings survived FDR correction in any model. No dorsal or median raphe-associated metabolic covariance findings survived FDR correction in any model. All p values were derived from 20,000 two-sided permutations.
